# Association between Social Network Characteristics and Lifestyle Behaviours in Adults at Risk of Diabetes and Cardiovascular Disease

**DOI:** 10.1371/journal.pone.0165041

**Published:** 2016-10-31

**Authors:** Sandra D. Bot, Joreintje D. Mackenbach, Giel Nijpels, Jeroen Lakerveld

**Affiliations:** 1 Department of General Practice and Elderly Care, EMGO Institute for Health and Care Research, VU Medical Centre Amsterdam, Amsterdam, the Netherlands; 2 Department of Epidemiology and Biostatistics, EMGO Institute for Health and Care Research, VU Medical Centre Amsterdam, Amsterdam, the Netherlands; National Institute for Viral Disease Control and Prevention, CHINA

## Abstract

**Objectives:**

In this exploratory study we examined the associations between several social network characteristics and lifestyle behaviours in adults at increased risk of diabetes and cardiovascular diseases. In addition, we explored whether similarities in lifestyle between individuals and their network members, or the level of social support perceived by these individuals, could explain these associations.

**Methods:**

From the control group of the Hoorn Prevention Study, participants with high and low educational attainment were approached for a structured interview between April and August 2010. Inclusion was stopped when fifty adults agreed to participate. Participants and a selection of their network members (e.g. spouses, best friends, neighbours, colleagues) completed a questionnaire on healthy lifestyle that included questions on fruit and vegetable intake, daily physical activity and leisure-time sedentary behaviour. We first examined associations between network characteristics and lifestyle using regression analyses. Second, we assessed associations between network characteristics and social support, social support and lifestyle, and compared the participants’ lifestyles to those of their network members using concordance correlation coefficients.

**Results:**

Fifty adults (50/83 x 100 = 62% response) and 170 of their network members (170/192 x 100 = 89% response) participated in the study. Individuals with more close-knit relationships, more friends who live nearby, and a larger and denser network showed higher levels of vegetable consumption and physical activity, and lower levels of sedentary behaviour. Perceived social norms or perceived support for behavioural change were not related to healthy lifestyle. Except for spousal concordance for vegetable intake, the lifestyle of individuals and their network members were not alike.

**Conclusions:**

Study results suggest that adults with a larger and denser social network have a healthier lifestyle. Underlying mechanisms for these associations should be further explored, as the current results suggest a minimal role for social support and modelling by network members.

## Introduction

Obesity, cardiovascular disease (CVD), type 2 diabetes, and several types of cancer can be prevented through a lifestyle with regular physical activity, low levels of sedentary behaviour and a balanced diet[1]. Higher fruit and vegetable consumption, as part of a dietary pattern that includes less energy dense foods and higher intakes of fibre and associated micronutrients, has been linked to smaller weight gain over time[2], several types of cancer[3] and lower risk of cardiovascular mortality[4]. The health benefits of regular physical activity are also irrefutable, with risk reductions of at least 20%-30% for more than 25 chronic medical conditions and premature mortality[5]. Sedentary behaviour, defined as any waking behaviour in a sitting or reclining position and a low energy expenditure[6], has been associated with all-cause mortality, independent of physical activity behaviour[7,8].

Despite these well-known benefits of a healthy lifestyle[9], adherence to guidelines for dietary intake and physical activity is limited[10]. As such, numerous interventions have been developed to improve lifestyle behaviours, especially for individuals at risk for type 2 diabetes and CVD. Such interventions are often based on theories such as the Health Belief Model[11], Theory of Planned Behaviour[12] and Social Cognitive Theory[13], focusing on individual-level psychological determinants of behaviour change[14]. Unfortunately, most interventions have limited and often temporary effects [15–18].

During recent years, socio-ecological models of health behaviour have gained recognition, suggesting that individual lifestyle is determined not only by individual-level factors but also by environmental-level–or ‘upstream’—factors[19]. One possible explanation for the limited long-term effects of lifestyle interventions may be that upstream drivers of lifestyle, such as social-structural conditions, are rarely included. This is despite a considerable evidence base supporting a link between the social environment, such as worker’s unions, sport associations and social support networks, and individual behaviour [20].

The importance of social networks—social structures composed of interdependent individuals; e.g. spouses, relatives, colleagues, neighbours, and friends[21]–for health has been recognized for decades. Several studies have shown that relatives and friends can play an important role in the promotion of healthy lifestyle behaviours, including for smoking cessation and reduction in alcohol intake [22–24]. A small number of studies have empirically linked social network characteristics with levels of physical activity and dietary behaviours. For example, the size of one’s social network has been positively associated with physical activity [25–27], and fruit and vegetable consumption [25].

Two underlying pathways for the relation between a social network and a healthy lifestyle have been proposed: i) similarities in lifestyle behaviours between individuals and their network members and ii) social support received from the network for behaviour change [28–30]. First, individuals may befriend peers with a similar lifestyle and subsequently friends may emulate each other’s behaviours. However, it is unclear whether convergence in behaviour may arise because persons form relationships with peers who have similar behaviours (i.e. selection processes) or because they change their behaviours to emulate their friends (i.e. influence processes)[28]. Second, a social network could provide support and resources for the start or maintenance of a healthy lifestyle [29]. For instance, network members may offer companionship as well as instrumental and emotional assistance; they can provide information about a healthy diet or exercise and at the same time emphasize the risks of unhealthy lifestyle behaviours[31]. For adolescents, literature suggests that social support may have an impact on lifestyle regardless of the lifestyle of the person providing support [30], and this may also be true for adults.

Few studies have investigated the importance of social networks for a healthy lifestyle or the conceptual pathways underlying this potential association. The findings from this study could be of use for designing primary prevention strategies in which individuals’ social networks are taken into account. In this study we investigated the link between several social network characteristics and fruit and vegetable intake, physical activity, and sedentary behaviour using the personal network approach. A personal network is a social network from the perspective of the individual at its centre [32]. We examined the moderating effect of gender and educational status, as previous studies suggested that women and individuals with higher education have larger networks [33–35], and that the association between social networks and health may differ by gender and educational status [36–39].

The aim of this exploratory study was to investigate the association between social network characteristics and lifestyle behaviours in adults at risk of type 2 diabetes and CVD. Although previous studies have mostly been conducted in healthy populations, we expect similar associations in a population at risk. This study provided the opportunity to explore whether similarities or differences between the lifestyles of individuals and their network members, or the social support received from the network as a whole, are potential underlying pathways explaining associations between social network characteristics and lifestyles in adults. We hypothesized that having a larger personal network, with more friends living nearby and more intense relationships, is related to healthier lifestyle behaviours. We also hypothesized that receiving social support from this network–regardless of whether the social support is specifically aimed at lifestyle–is associated with healthier lifestyle behaviours, and that the lifestyles of individuals and their network members are alike.

## Materials and Methods

### Study population

Participants for the current study were recruited from the control group of the Hoorn Prevention Study. Details about the Hoorn Prevention Study are provided elsewhere [40]. Briefly, contact details of men and women aged 30–50 years, living in the semi-rural region of West-Friesland, the Netherlands were derived from computerized databases of the participating general practices (n = 12). All individuals in the Netherlands are registered with a general practice. Eligible participants were screened for risk of diabetes and CVD based on self-measured waist-circumference and the ARIC (Atherosclerosis Risk In Communities) score [41] and randomized into the intervention or control group. The control group of the Hoorn Prevention Study consisted of 308 persons aged 30–50 years, of which 59 participants (19%) had a high education level (college, university) and 103 participants (33%) a low education level (primary school or less). Participants from the control group were approached for a structured interview between April and August 2010. Inclusion was stopped when fifty adults agreed to participate. The study was approved by the Medical Ethics Committee of the VU University Medical Center in Amsterdam and all participants gave written informed consent.

### Interviews

Structured interviews of up to one hour’s duration were held by an experienced interviewer at the Diabetes Research Centre in Hoorn. Social networks of the participants were reconstructed using the *exchange relationship Name Generator / Interpreter* method [42]. In this method, the names of relevant network members are systematically obtained by using the following name generating (NG) questions: ‘Do you know anyone: NG1) who gives advice on problems at work?; NG 2) to whom do you give advice regarding problems at work?; NG 3) with whom do you work together often?; NG 4) who helps you with small jobs around the house?; NG 5) who keeps a spare key to your house?; NG 6) who is your direct neighbour?; NG 7) who do you visit for social contact?; NG 8) with who do you talk about important matters?; NG 9) who is important to you besides those mentioned in response to the previous questions?

All persons mentioned were listed on a printed response matrix. For most name generating questions, we limited the number of names that could be mentioned to 5. Previous research has shown that some relationships are particularly prone to be forgotten in interview situations [43], therefore the list was shown to the participant to ensure that additional important relationships were not forgotten. In the three preceding pilot interviews, we discovered that the participants often forgot to mention their partner, children or parents, therefore we decided to add the following question to the list (NG 10): are there any important family members, who have not been mentioned previously? Relationship attributes measured and used for this study were: perceived role relationships (e.g. partner, friend, neighbour, colleague), perceived relationship intensity, and geographic nearness (i.e. living within a distance of 5 km).

### Network members

At the end of the interview, participants were asked to give a selection of all network members on the list (i.e. spouse, best friend, parent, sibling, colleague, and neighbour), whom were subsequently sent a questionnaire to assess daily leisure time physical activity, fruit and vegetable intake, and leisure time sedentary behaviour.

#### Dependent variables–lifestyle behaviours

Outcome measures were retrieved from measurements of the Hoorn Prevention Study:

- Self-reported physical activity was assessed by the SQUASH, a validated questionnaire to measure daily physical activity [44]. The SQUASH gives an indication of the daily habitual activity level per day of physical activity in minutes per day.
- Leisure time sedentary behaviour, measured with the Activity Questionnaire for Adults and Adolescents (AQuAA) [45], including questions about amount of time watching TV, using the computer, reading, and other sedentary activities such as sitting at a bar, playing board games, and driving a car. We used daily MET-minutes leisure time sedentary behaviour, representing the time engaged in leisure time sedentary behaviour multiplied by the metabolic equivalent value.
- Fruit intake (pieces per day) and vegetable intake (grams per day) were assessed by an extended and modified version of the 8-item Food Frequency Questionnaire [46].

#### Independent variables–social network characteristics

Network size, a measure counting the total number of people mentioned in response to all network delineating questions.

- Network density is the number of existing relationships divided by the maximum number of relationships in the network. The relationships between persons first named in response to name generating questions NG1, NG2, NG4, NG5, NG7, and NG8 were investigated. Participants indicated whether pairs of network members (1) avoided each other, (2) did not know each other (3) hardly know each other (4) know each other well, or (5) know each other well and get along very well. These values were dichotomised into “0”: avoiding each other, not knowing each other, and hardly knowing each other; and “1”: knowing each other well, and getting along very well. Network density was calculated as the number of relationships coded 1, divided by the total number of network member pairs mentioned (range 0–1; with 1 indicating a dense network in which everybody knows each other well)[47].
- Number of relatives and friends living nearby, a measure counting the number of relatives and friends living within a distance of 5 km of the participant.
- Number of intense relationships, a measure counting the number of relationships of which the participant selected the maximum score on the question how intense the relationship with the network member is on a 5-point scale. In this study, intense relationships were mostly with family members (85%) and friends.

#### Mediating variables–social support

We used the following two variables as indicators of social support:

- Perceived influence of friends on health behaviours When a ‘best friend’ was mentioned by the participant, the interviewer asked further questions to assess how much influence he/she has on the participant’ s health behaviours (1 (no influence) to 5 (a lot of influence)). High perceived influence of friends was given a score of 4 or 5.
- Perceived norms of network members towards healthy behaviours. Perceived norms regarding increasing physical activity and eating healthier food was measured with the Determinants of Lifestyle Behavior Questionnaire [48]. This is a valid instrument for measuring theory of planned behaviour constructs in relation to diet and physical activity behaviours in adults at high risk of type 2 diabetes and cardiovascular diseases [48]. Perceived norms were measured with items stating ‘My partner/family/friends think(s) that I should be more physically active/ eat healthier food on a 5-point Likert-type scale ranging from 1 (totally disagree) to 5 (totally agree). Overall mean scores for the participants’ network members were calculated separate for physical activity and dietary behaviour. Scores from 0–3 were considered low and scores >3 were considered high.

### Statistical methods

Descriptive statistics (means ± SD, or median and interquartile ranges, as appropriate) were used to describe the study sample and their network characteristics, as well as the characteristics of the network members. Multiple linear and logistic regression analyses were carried out, adjusted for sex and education. Possible effect-modification by sex or education was examined by including an interaction term in the model. In case of significant effect modification (P<0.1), stratified analyses were carried out. To test for mediation by social support, simple regression analyses were conducted to examine the associations of social network characteristics with social support, and of social support with lifestyle behaviours. Residual histograms and residual plots were used to check the linearity of the regression models. Right skewed data was log-transformed if that improved normality of the data prior to the statistical analyses. Agreement between participants’ lifestyle behaviours with those of their network members was measured using Lin’s concordance correlation coefficient (CCC). Lin’s CCC is the Pearson coefficient of correlation, which assesses the closeness of the data to the line of best fit, modified by taking into account how far the line of best fit is from the 45- degree line through the origin[49]. Statistical tests were two-sided and significance was set at a value of *P*-values below 0.05. All analyses were conducted using SPSS 22.0 (SPSS Inc., Chicago, IL, USA), except for Lin’s CCCs, which were analysed in R statistical software (version 3.2.2).

## Results

Eighty-six people were sent an invitation letter to participate in this study, of whom 53 agreed to participate and 50 were interviewed (28 declined to participate, 5 people could not be contacted and it was not possible to schedule a meeting for three individuals). This resulted in a 62% response rate. In total, 192 selected network members were sent a questionnaire and 170 were completed (89% response).

Baseline characteristics of the study population (for the total population as well as by sex and education separately) and characteristics of their social networks are presented in Table 1. Men and individuals with low education had higher levels of total physical activity. Vegetable intake was higher among individuals with high education. The size of women’s social networks was larger than of men’s social networks, and individuals with high education had larger social networks than individuals with low education. Individuals with low education had more network members living nearby than individuals with high education.

**Table 1:** Socio-demographic characteristics of the study participants.

	Total (n = 50)	Men (n = 22)	Women (n = 28)	High education (n = 24)	Low education (n = 26)
**Sex**–female, n (%)	28 (56%)	xxx	xxx	16 (67%)	12 (46%)
**Age**–yr, mean (SD)	46.6 (5.3)	46.2 (5.0)	47.0 (5.6)	45.2 (6.3)	47.9 (3.9)
**Employed–**n (%)	43 (86%)	22 (96%)	21 (79%)	21 (88%)	22 (85%)
**Education (low)–**n (%)	26 (52%)	14 (64%)	12 (43%)	xxx	xxx
**Income (below average)–**n (%)	4 (8%)	2 (9%)	2 (7%)	3 (12%)	6 (23%)
**Married / living together**–n (%)	43 (86%)	18 (82%)	25 (89%)	21 (88%)	22 (85%)
**Body mass index–**mean (SD)	28.9 (4.8)	28.7 (4.6)	29.0 (5.0)	29.3 (5.1)	28.5 (4.5)
**Smoking**–n (%)	7 (14%)	5 (23%)	2 (7%)	2 (8%)	5 (19%)
**Total physical activity**^**a**^ **–**mean (SD)	397.6 (165.1)	411.1 (196.5)	387.2 (138.4)	360.0 (143.0)	432.3 (178.8)
**Leisure time sedentary behaviour**^b^ ^–^median (Q1; Q3)	298.9 (219.1; 380.1)	283.9 (198.5; 386.8)	303.2 (229.8; 376.9)	292.5 (205.7; 382.5)	304.3 (238.7; 380.1)
**Fruit intake–**pieces per day, median (Q1; Q3)	1.0 (0.4; 2.0)	1.1 (0.4; 2.0)	1.0 (0.5; 2.0)	0.9 (0.4; 1.9)	1.4 (0.5; 2.0)
**Vegetable intake–**grams per day, median (Q1; Q3)	150.0 (100.0; 185.7)	146.4 (107.1; 191.1)	150.0 (85.7; 178.6)	157.1 (121.4; 200.0)	132.1 (89.3; 171.4)
**Network size–**mean (SD)	20.2 (6.1)	18.6 (4.9)	21.4 (6.7)	21.7 (6.3)	18.7 (5.6)
**Number of friends–**mean (SD)	4.1 (3.2)	3.1 (2.6)	4.8 (3.5)	4.3 (3.4)	3.8 (3.1)
**Network density–**mean (SD)	0.4 (0.2)	0.4 (0.2)	0.5 (0.2)	0.4 (0.2)	0.5 (0.2)
**Number of relatives & friends < 5 km away–**mean (SD)	7.9 (4.9)	7.1 (2.7)	8.5 (6.1)	6.9 (3.8)	8.8 (5.7)
**Number of intense relationships–**mean (SD)	6.4 (2.9)	6.7 (2.6)	6.1 (3.2)	6.4 (2.8)	6.4 (3.1)
**High perceived norms for changing physical activity–**n (%)	19 (39%)	11 (50%)	8 (30%)	12 (52%)	7 (27%)
**High perceived norms for eating healthier–**n (%)	13 (21%)	6 (29%)	7 (26%)	8 (36%)	5 (19%)
**High perceived influence of friends on lifestyle–**n (%)	9 (26%)	3 (25%)	6 (26%)	8 (42%)	1 (6%)

^a^ Minutes per day engaged in physical activity

^b^ MET-minutes per day during leisure time

n for some variables is reduced due to missing data

The socio-demographic characteristics of the total sample of social network members, as well as by type of relationship, are presented in Table 2. Partners and best friends were the most frequently listed network members (21% of all network members were partners and 21% were best friends). Siblings and neighbours on average has highest levels of total physical activity, while children had the lowest levels. Parents and siblings consumed highest levels of vegetables.

**Table 2:** Socio-demographic characteristics of network members.

	Total^a^ (n = 170)	Partner (n = 35)	Parent (n = 23)	Sibling (n = 20)	Child (n = 8)	Best friend (n = 35)	Colleague (n = 14)	Neighbour (n = 20)
**Sex**–female, n (%)	104 (61%)	14 (41%)	17 (34%)	11 (55%)	5 (71.4%)	23 (66%)	5 (36%)	13 (65%)
**Age**–yr, mean (SD)	51.1 (13.3)	47.7 (6.0)	71.8 (7.4)	48.4 (8.2)	22.3 (8.9)	47.9 (3.9)	46.9 (8.7)	49.9 (7.5)
**Education (low)–**n (%)	13 (8%)	1 (2.9%)	7 (38.1)	1 (5%)	2 (25%)	1 (3%)	0 (0%)	0 (0%)
**Income (below average)–**n (%)	33 (19%)	3 (8.8%)	8 (38.1)	3 (15.8%)	2 (25%)	9 (26%)	2 (14%)	3 (15%)
**Total physical activity**^b^ **–**mean (SD)	375.4 (137.8)	394.4 (134.5)	231.9 (150.3)	424.3 (203.6)	187.8 (131.1)	399.0 (209.1)	379.4 (148.0)	428.9 (142.8)
**Leisure time sedentary behaviour**^c^ ^–^median (Q1; Q3)	296.5 (242.5; 366.3)	257.1 (151.9; 312.3)	390.5 (80.9; 334.3)	259.3 (183.5; 334.0)	416.8 (228.5; 787.5)	275.9 (227.9; 394.8)	289.3 (196.1 316.1)	286.1 (214.3; 377.7)
**Vegetable intake–**grams per day, median (Q1; Q3)	161.9 (132.5; 212.2)	128.6 (85.7; 171.4)	171.4 (114.3; 225.0)	160.7 (114.3; 208.9)	121.4 (107.1;169.6)	150.0 (103; 242.9)	146.4 (100; 282.1)	153.6 (123.1; 191.1)

n for some variables is reduced due to missing data

^a^15 network members were only analysed in the total group; their type of relation with the participant was classified as ‘miscellaneous’

^b^Minutes per day engaged in physical activity

^c^MET-minutes per day during leisure time

Associations between the network characteristics and the different outcomes (adjusted for sex and education) are shown in Table 3. As interaction terms indicated that for some associations there was significant effect modification by gender, these associations are presented stratified. Having a larger network was associated with more physical activity and less sedentary behaviour during leisure time: each additional network member was associated with an additional 9 minutes of physical activity per day for women (but not for men), and 8 minutes per day less sedentary behaviour.

**Table 3:** Associations between lifestyle behaviours and network characteristics.

	Total physical activity^a^	Leisure time sedentary behaviour^b^	Fruit intake (pieces/day)	Vegetable intake (grams/day)
**Network size**	women 9.3 (1.5 to 17.1)*	-8.4 (-16.7 to -0.2)*	0.02 (-0.03 to 0.06)	0.9 (-3.1 to 4.9)
(per increase of 1 member)	men -4.1 (-22.6 to 14.4)			
**Number of friends**	women 16.9 (2.2 to 31.6)*	-8.9 (-24.7 to 6.9)	0.01 (-0.08 to 0.09)	4.0 (-3.6 to 11.6)
(per increase of 1 friend)	men -2.7 (-39.9 to 34.5)			
**Network density**	2.8 (-20.0 to 25.6)	women -4.9 (-29.5 to 39.3)	0.01 (-0.11 to 0.13)	4.3 (-6.7 to 5.3)
(per 0.1 increase)		men -40.1 (-75.9 to -4.3)*		
**Number of relatives and friends < 5 km away**	14.6 (5.5 to 23.6)*	-4.4 (-15.8 to 6.8)	0.04 (-0.02 to 0.09)	-2.8 (-7.6 to 2.1)
**Number of intense relationships**	-1.6 (-18.0 to 14.7)	-4.6 (-21.5 to 12.4)	0.02 (-0.06 to 0.11)	8.2 (0.6 to 15.7)*

Values are regression coefficients (95% confidence interval) adjusted for sex and education

*p < 0.05

^a^Minutes per day engaged in physical activity

^b^MET-minutes per day engaged in sedentary behaviours

^c^We linked the item ‘high norms towards increased physical activity’ to physical activity and sedentary behaviour, and the item ‘high norms towards increased healthy eating’ to fruit and vegetable intake

In women, the number of friends was associated with more time spent doing physical activity: each additional friend was associated with an extra 17 minutes of total physical activity daily.

For men, a higher network density was associated with less sedentary behaviour.

Having more relatives and friends living nearby (within 5 kilometres) was associated with more total physical activity: one additional relative or friend living close by was associated with an additional 15 minutes of total physical activity per day.

The number of intense relationships (i.e. the number of network members on the list to whom the participant allocated the maximum intensity score) was associated with greater vegetable intake: each additional intense relationship was associated with an increased vegetable intake of 8 grams per day.

Tables 4 and 5 display the mediating pathway of social support in the association between network characteristics and lifestyle behaviours. Table 4 shows that there were no statistically significant associations between network characteristics and social support. Table 5 presents the associations between social support and lifestyles. Neither high perceived norms towards lifestyle change, nor high perceived influence of friends on lifestyle, were significantly associated with any of the lifestyle factors.

**Table 4:** Association between network characteristics and social support (first step mediation analyses).

	High perceived norms towards eating healthier	High perceived norms towards increased physical activity	High perceived influence of friends on lifestyle
**Network size** (per increase of 1 member)	1.01 (0.90 to 1.13)	1.00 (0.89 to 1.11)	1.09 (0.92 to 1.28)
**Number of friends** (per increase of 1 friend)	1.02 (0.83 to 1.25)	0.85 (0.67 to 1.09)	1.16 (0.89 to 1.51)
**Network density** (per 0.1 increase)	0.95 (0.69 to 1.32)	0.89 (0.65 to 1.23)	1.14 (0.78 to 1.66)
**Number of relatives and friends <5 km away**	1.10 (0.96 to 1.26	1.06 (0.93 to 1.22)	0.91 (0.73 to 1.14)
**Number of intense relationships**	0.81 (0.62 to 1.06)	0.80 (0.62 to 1.03)	0.87 (0.63 to 1.22)

Values are Odds ratios (95% confidence interval) adjusted for sex and education

p < 0.05 (but none of the associations were statistically significant)

**Table 5:** Associations between social support and lifestyles (second step mediation analyses).

	Total physical activity^a^	Leisure time sedentary behaviour^b^	Fruit intake (pieces/day)	Vegetable intake (grams/day)
**High perceived norms towards lifestyle change**^c^	-28.1 (-134.4 to 78.2)	-67.3 (-175.7 to 41.1)	-0.27 (-0.86 to 0.32)	-40.5 (-94.1 to 13.1)
**High perceived influence of friends on lifestyle**	-88.4 (-216.1 to 39.2)	-34.4 (-159.6 to 90.8)	-0.36 (-1.08 to 0.36)	-45.6 (-108.4 to 17.2)

Values are regression coefficients (95% confidence interval) adjusted for sex and education

p < 0.05 (but none of the associations were statistically significant)

^a^Minutes per day engaged in physical activity

^b^MET-minutes per day engaged in sedentary behaviours

^c^We linked the item ‘high norms towards increased physical activity’ to physical activity and sedentary behaviour, and the item ‘high norms towards increased healthy eating’ to fruit and vegetable intake

Lin’s CCCs of the lifestyle behaviours of participants and network members ranged from r = -0.23 to r = 0.42, indicating weak or no agreement between the lifestyle behaviours, Only Lin’s CCC of the participant’s vegetable intake and his/her partner’s vegetable intake (0.42; 95%CI 0.12; 0.65) was statistically significant. Results are presented in Table 6.

**Table 6:** Concordance between participants’ and network members’ lifestyle behaviours.

	Total physical activity^a^	Leisure time sedentary behaviour^b^	Vegetable intake (grams/day)
**Partner** (n = 35)	-0.02 (-0.33; 0.30)	0.11 (-0.17; 0.37)	0.42 (0.12; 0.65)*
**Parent** (n = 23)	0.09 (-0.19; 0.36)	0.26 (-0.10; 0.57)	0.23 (-0.06; 0.48)
**Sibling** (n = 20)	-0.23 (-0.59; 0.20)	0.08 (-0.33; 0.47)	-0.02 (-0.36; 0.33)
**Child** (n = 8)	0.07 (-0.28; 0.40)	-0.02 (-0.24; 0.21)	0.26 (-0.42; 0.75)
**Best friend** (n = 35)	0.16 (-0.16; 0.45)	-0.02 (-0.35; 0.31)	0.19 (-0.16; 0.50)
**Colleague **(n = 14)	0.14 (-0.41; 0.62)	-0.16 (-0.58; 0.33)	0.06 (-0.33; 0.44)
**Neighbour** (n = 20)	-0.23 (-0.58; 0.19)	-0.01 (-0.44; 0.43)	0.00 (-0.42; 0.42)

Values are Lin’s concordance correlation coefficients (95% confidence interval) and ranges from -1 to 1, with perfect concordance at 1.

* p < 0.05.

^a^Minutes per day engaged in physical activity

^b^MET-minutes per day engaged in sedentary behaviours

## Discussion

In this exploratory study we provided evidence for a relation between the size and density of an individual’s social network and lifestyle behaviour. In line with our hypothesis, we found a number of small, yet statistically significant associations between lifestyle behaviours and network size, network density, number of friends, number of relatives and friends nearby and number of intense relationships. In general, the associations were in the expected directions, such that a larger, denser network, more friends living nearby and more intense relationships were associated with healthier behaviours. We found evidence that gender modified some of the associations. Network characteristics were more strongly related to increased physical activity in women, and more strongly related to decreased sedentary behaviour in men. We found no evidence for a moderating effect of educational status. Our findings did not support the hypothesis that receiving social support from or having similar lifestyle behaviours as your network members mediates the relation between social network characteristics and lifestyle behaviours. However, there was a lack of agreement between the lifestyle behaviours of individuals and that of members of their network. Despite the relatively high response rate among participants (62%) and their network members (89%), the sample sizes did not allow for a more detailed assessment of the interrelation between participants’ and network members’ lifestyle behaviours.

In line with our findings, some studies have found positive associations between the number of close relations and physical activity [26,27]. Among low income adults living in public housing, residents with small social networks took fewer steps a day compared to residents with larger social networks [26]. In a population based health study, the number of close relatives and close friends was weakly but significantly associated with higher levels of leisure time physical activity [27]. Moreover, the likelihood of physical activity was found to be higher in those with larger social networks and more perceived social support [25]. One of the mechanisms through which social network members may influence each other is by providing social support that enables health promoting behaviours [50,51]. For example, social support from family and friends has consistently been found to be positively correlated with physical activity [52]. In the present study however, there was no evidence that network members supported healthy lifestyle, nor was the perceived influence of friends on lifestyle associated with physical activity (both proxies of social support). One possible explanation may be that only 40% of individuals reported receiving high social support from their friends.

Social network characteristics were mainly associated with physical activity and sedentary behaviour outcomes, and less so with fruit and vegetable intake. Only the number of intense relationships was associated with increased vegetable intake. This contrasts with studies indicating that an increased social network size was associated with an increase in combined fruit and vegetable intake [53]. However, social network size had another definition than we used in our study. Although social support and social norms have been shown strong predictors for fruit and vegetable intake [33,51,52], they were not associated with vegetable intake in this study. It may be that fruit and vegetable intake is mainly influenced by family members at home and less by friends, relatives and colleagues. No other studies related social networks to sedentary behaviours, although activities with peers are likely to be sedentary: having dinner, playing board games, watching a movie, going out for drinks. Surprisingly, the present study found that among women, denser networks were associated with less leisure time sedentary behaviour.

Such reverse associations have been observed in previous studies as well, such that larger or denser social networks negatively affect healthy lifestyles. For example, Christakis & Fowler found an increased risk of becoming obese if a spouse, sibling or friend became obese [22]. This suggests that social network characteristics could be enhancing or impeding for the adoption and maintenance of healthy lifestyle behaviours.

Except for vegetable intake, we found no similarities in the participants’ lifestyle and that of their network members. Previous research has demonstrated that family members' eating habits are similar [54] and that spouses show the strongest concordance in eating patterns over time [55]. Although we did not find such similarities in physical activity or sedentary behaviour, a Portuguese study showed that correlations in physical activity were stronger between spouses than between parents and offspring, with effect sizes comparable to our study [56].

This study has some limitations. Due to the cross-sectional design, we were unable to attribute causality to the associations found. For instance, the association between network size and physical activity could also be explained by the fact that being physically active (e.g. in organised exercise activities) consequentially leads to a larger social network. Next, compared to self-reported questionnaires, reconstructing social networks and interviewing participants is a costly and time-consuming process and resulted in a relatively small sample size. Consequently, some associations found may be false positive, and some important associations may not have reached statistical significance. In addition, the small sample size prevented us from adjusting for other possible confounders such as age, smoking or body weight (although it was an age-homogeneous population), or the formal exploration of mediating pathways. Lastly, a disadvantage of using Lin’s CCC to assess the concordance between participants’ and network members’ lifestyle behaviours is the inability to adjust for confounding variables. On the other hand, the face-to-face interviews allowed us to collect detailed and complete information about the participants’ personal network. A unique strength of our study was the linkage between participants’ lifestyle behaviours and network members’ lifestyle behaviours, which allowed us to assess similarity in lifestyle. Finally, information on network members’ lifestyle behaviours allowed us to compare the lifestyle behaviours of individuals and their network members.

In summary, this study presents empirical evidence for the relation between several social network characteristics and lifestyle behaviours in adults at risk of type 2 diabetes and CVD. Lifestyle behaviours are influenced by many factors, and our findings confirm the importance of social-structural conditions. The density, size and proximity of one’s social network may be important factors that have not received a lot of attention yet. The current results from this exploratory study suggest a minimal role of social support and modelling, but underlying pathways should be further explored in studies that are powered to do so.

## Supporting Information

S1 Dataset Dataset Social Networks PLoS. (XLSX)
